# Designed to Fail? Revisiting Uganda’s Maternal Health Policies to Understand Policy Design Issues Underpinning Missed Targets for Reduction of Maternal Mortality Ratio (MMR): 2000-2015

**DOI:** 10.34172/ijhpm.2021.127

**Published:** 2021-09-07

**Authors:** Moses Mukuru, Jonathan Gorry, Suzanne N. Kiwanuka, Linda Gibson, David Musoke, Freddie Ssengooba

**Affiliations:** ^1^Department of Health Policy, Planning and Management, School of Public Health, College of Health Sciences, Makerere University, Kampala, Uganda.; ^2^School of Social Sciences, Nottingham Trent University, Nottingham, UK.; ^3^Department of Disease Control and Environmental Health, School of Public Health, College of Health Sciences, Makerere University, Kampala, Uganda.

**Keywords:** Uganda, Maternal Mortality, Policy Mixes, Three Delay Model, Policy Design

## Abstract

**Background:** Despite Uganda and other sub-Saharan African countries missing their maternal mortality ratio (MMR) targets for Millennium Development Goal (MDG) 5, limited attention has been paid to policy design in the literature examining the persistence of preventable maternal mortality. This study examined the specific policy interventions designed to reduce maternal deaths in Uganda and identified particular policy design issues that underpinned MDG 5 performance. We suggest a novel prescriptive and analytical (re)conceptualization of policy in terms of its fidelity to ‘3Cs’ (coherence of design, comprehensiveness of coverage and consistency in application) that could have implications for future healthcare programming.

**Methods:** We conducted a retrospective study. Sixteen Ugandan maternal health policy documents and 21 national programme performance reports were examined, and six key informant interviews conducted with national stakeholders managing maternal health programmes during the reference period 2000-2015. We applied the analytical framework of the ‘three delay model’ combined with a broader literature on ‘policy mixing’.

**Results:** Despite introducing fourteen separate policy instruments over 15 years with the goal of reducing maternal mortality, by the end of the MDG period in 2015, only 87.5% of the interventions for the three delays were covered with a notable lack of coherence and consistency evident among the instruments. The three delays persisted at the frontline with 70% of deaths by 2014 attributed to failures in referral policies while 67% of maternal deaths were due to inadequacies in healthcare facilities and trained personnel in the same period. By 2015, 37.3% of deaths were due to transportation issues.

**Conclusion:** The piecemeal introduction of additional policy instruments frequently distorted existing synergies among policies resulting in persistence of the three delays and missed MDG 5 target. Future policy reforms should address the ‘three delays’ but also ensure fidelity of policy design to coherence, comprehensiveness and consistency.

## Background

**Key Messages**

** Implications for policy makers**

- Ugandan maternal mortality in the period 2000-2015 corresponds to ‘three delays’ – ie, driven by the interplay between delayed action at home, transportation and appropriate healthcare within and between health facilities.
- If not well designed, the adoption of new policy instruments can distort synergies across existing policies and create a policy mix that is not coherent, consistent nor comprehensive enough to impact maternal mortality ratio (MMR).
- Given the Sustainable Development Goals (SDGs) inherited some policies from the Millennium Development Goal (MDG) period, policy-makers should carefully consider re-examining the current policy mix for maternal health to ensure their alignment and collective impact on the ‘three delays’ responsible for maternal death.

** Implications for the public**

Reducing maternal mortality requires a multi-sectoral approach that increases education about, and access to, functioning emergency obstetrics. Concurrent operation of policy packages with contradictory foci like the case was in basic universal, comprehensive and selective high impact policies, can render policy reforms ineffective. Addressing such contradictions among policy packages should be one of the goals of policy reform. Therefore, while policy-makers should reform the current policies to address the design flaws and accelerate the reduction in maternal mortality ratio (MMR), care should be taken to ‘join-up-thinking’ and strengthen existing synergies among the policy instruments. The 3Cs (coherence of design, comprehensiveness of coverage and consistency in application) framework proposed in this paper is a useful tool for guiding this process.

Few would dispute there are many social, economic and political factors at play when trying to understand why maternal mortality ratios (MMRs) did not decline sufficiently to meet Millennium Development Goals (MDGs) targets.^1,2^ The more insurmountable issues involve navigating well-trodden paths that regard the endemic nature of poverty, associated health issues of pregnant women, or more open-ended and difficult to influence discussions regarding investment in health services and more specifically maternal health.^3-5^ Notwithstanding the challenging context, this paper momentarily shifts gaze away from what can be considered the *uncontestable* ‘technical’ details about maternal health, to the more *contestable* technical details regarding the actual policies politicians and policy-makers introduced to alleviate maternal mortality. We are not arguing that looking through the lens of policy design is the only answer to a myriad of problems but rather it offers a different and creative perspective in looking at health policy. By examining maternal health policies introduced to tackle the high MMR in Uganda during the MDG period albeit their suboptimal performance, we hope to deconstruct policy design issues that underpin policy performance.

 Despite the centrality of policy design to performance (and its pre-eminence in the policy sciences literature), this theme has received surprisingly limited attention from studies on maternal health.^6^ According to Caprano and Howlett, policy design refers to the mixture of “instruments expected to more or less comprehensively attain a set of goals.”^7^ The literature on policy design is important because extant studies have at least partly attributed the persistence of high mortality to distortions in maternal health priorities in particular the failure to deliver exhaustive or ‘universal’ policy packages.^8,9^ One way to understand policy design is by reflecting on Thaddeus and Maine’s three-delay framework (hereafter 3D) which was introduced in 1994 and quickly impacted maternal health studies in low- and middle-income countries.^10,11^ Various papers have applied this framework to explain the persistence of high mortality with much valuable research conducted in epidemiology, quality of care, coverage and so forth.^12-17^ This paper argues that although policy reforms undertaken in Uganda to reduce maternal mortality during the MDG period (2000-2015) were underpinned either explicitly or implicitly, by design or accident, in ways that reflect the 3D model as a theory of change, the MDG 5 targets of 131 deaths per 100 000 live births (a 75% reduction in the MMR) were missed and policy failure observed.^18^ Here we are particularly interested in drawing the attention of policy scholars and practitioners to the significance of ‘policy mixing’ to better understand failure to achieve targets. Policy mixing is “the extent to which multiple policy instruments, sequenced and assembled in portfolios or bundles, work in concert to give effect to different aspects of a policy goal.”^19^ Our consequent aim is to identify and then emphasise the importance of the ‘3Cs’ (coherence of policy design, comprehensiveness of policy coverage and consistency in policy application) within and across the various policy shifts to help explain missed targets. We answer two distinctive questions: (1) How did the evidence on the three delays framework manifest in the context of persistent maternal deaths in Uganda during the MDG period? (2) What were the contradictions and/or synergies in the design of the various maternal health policy packages actioned? The need to scrutinise the influence of policy design on maternal health outcomes is no less important with the close of MDGs and the onset of Sustainable Development Goals (SDGs). This paper ultimately seeks to inform policy reforms that could contribute to the achievement of SDG target 3.1 of less than 70 maternal death per 100 000 live births by 2030.

 In 1999, the Ugandan government published its ‘*National Health Policy*’ with the commitment to offering a Universal Minimum Healthcare Package (UNMHCP) commonly known as the ‘basic care package.’ Section 4.2.3 detailed the approach to ‘Sexual and Reproductive Health and Rights’ and ‘Essential Ante-natal and Obstetric Care’ as one of “…ensur[ing] safe pregnancy and delivery, improved management of complications of pregnancy and childbirth including spontaneous or induced abortion, and (to) *reduce the unacceptably high rates of maternal and perinatal deaths through timely and effective emergency obstetric care *[EmOC] *provided at strategic and accessible locations* (emphasis added).”^20^ In 2006/2007, a more focused ‘*Road Map for Accelerating the Reduction of Maternal and Neonatal Mortality*’ was introduced. The vision was “[t]o have women in Uganda go through pregnancy, childbirth and postpartum period safely, and their babies born alive and healthy.”^21^ The overall goal was to achieve the MDG target of 131 deaths per 100 000 live births through a ‘comprehensive policy package.’ The Road Map aimed to secure this in three ways:

1. By increasing the availability, accessibility and utilization of quality skilled care during pregnancy, childbirth and postnatal periods at all levels of the healthcare delivery system.
2. By promoting and supporting appropriate health seeking behaviour among pregnant women, their families and the community.
3. By strengthening family planning information and service provision for women/men/couples who want to space or limit their childbearing preventing unwanted and/or untimely pregnancies that increase the risk of maternal death.

Three years after the introduction of this ‘comprehensive package,’ the Ministry of Health (MoH) produced National Health Policy II – Uganda’s second national health policy – entitled *Promoting People’s Health to Enhance Socio-economic Development*. From 2010 the emphasis was now more on general health promotion, but the essential idea was ultimately to generate ‘universal’ delivery of the 1999 UNMHCP ‘basic package.’ In 2013 a new ‘Sharpened Plan for Uganda’ entitled *‘Reproductive Maternal, Newborn and Child Health*’ was unveiled as an evidence-based plan that recognised the likely failure to achieve MDG 5. From here the emphasis switched to selective or ‘high impact’ interventions.

 During the MDG period, Uganda undertook focussed, varied and often complex policy changes to reduce preventable maternal mortality. Three broad shifts in policy design can be observed: ‘basic’ (1999-2010); ‘comprehensive’ (2007); and ‘high impact’ (2013) packages.^20-22^ But these packages of policy instruments did not translate into their intended target of significant reduction in maternal mortality.^18^ According to the Uganda Demographic and Health surveys (UDHS) conducted between 2000 and 2016, Uganda experienced an insignificant reduction in the MMR. As noted, the stated MDG target was to reduce MMR by 75% (ie, 131 deaths per 100 000 per live births) by 2015, yet Uganda only managed to reduce fatalities by 30% (from 524 to 368 deaths per 100 000 live births).^23,24^ For two-thirds (10 years) of the MDG period, the main facilitators of effective maternal healthcare – deliveries in health facilities, skilled attendance at birth and attendance of the recommended four antenatal care visits (4th ANC), were on average performing at 50% or less as the MMR stagnated at 438 deaths per 100 000 live births.^25^ Although significant improvements were experienced in the final five years of the MDG period (health facility deliveries increased to 73%, skilled birth attendance to 74%, 4th ANC attendance to 60%) and the MMR declined to 368 deaths per 100 000 live births, missing the overall MDG target for maternal mortality.

## Methods

###  Conceptual Framework 

 This paper combines Thaddeus and Maine’s ‘three delays’ model with policy mix design approach.^19,26^ In the 3D model, Thaddeus and Maine convincingly argued that the prevention of maternal mortality is largely dependent on the length of the interval between the onset of obstetric complication and its outcome. Prompt and adequate treatment promotes positive outcomes while delayed treatment adversely affects outcomes. Delay I refers to delays in seeking care.^27,28^ Delay II involves delays in accessing a health facility with functional EmOC services; and Delay III involves the delay in accessing appropriate EmOC services once at the facility, or due to subsequent referral from one health facility to another (ie, delays related to correct diagnosis of complications and appropriate action and, delays in the referral pathways).^9,29-31^ According to 3D, the delays in seeking care, reaching a health facility and receiving appropriate care feed into each other. For example, the delay to seek care is affected by how far a pregnant woman has to travel for healthcare (Delay II) but also the mother’s perception of the quality of services in respective health facilities (Delay III). In addition, the three delays can occur in a vicious cycle with delay I leading to delay II, which in turn leads to delay III as the cycle continues.

**Figure 1:**
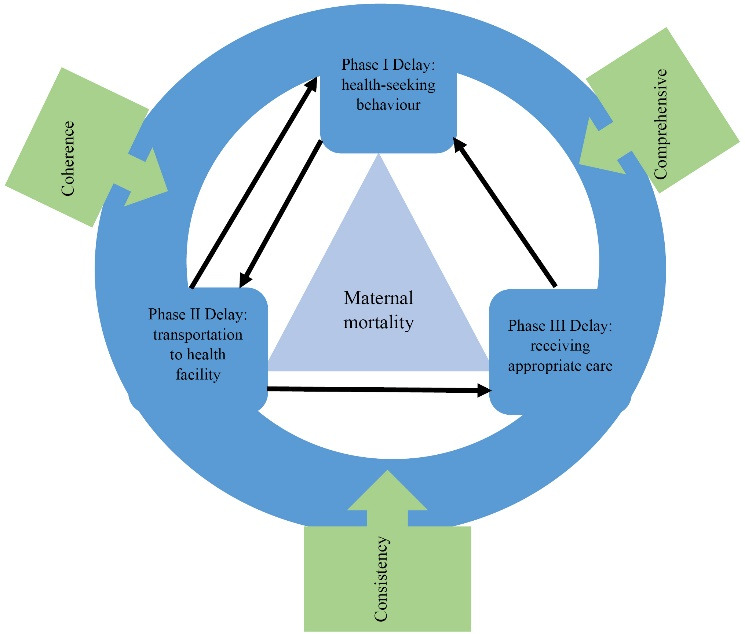
Conceptual Framework.

 The paper derives from the broader social science literature on policy design as understood in two principle ways: First, procedurally as a process of selecting the most appropriate policy instruments to solve a problem or achieve a policy goal,^19^ Second, as a substantive output of the policy process – the content, instruments and goals achieved.^32^ Policy instruments refer to “the means by which government policies are carried out.”^33^ Policies may well arise from an objective process of problem analysis and rational selection of appropriate instruments. However, they could also arise out of a negotiation or clash of interests.^34^ We position ourselves here as ‘rationally substantive’ where policy design is considered as an outcome of an objective process that combines policy instruments to achieve the goals sought. An effective policy design basically is one that is able to solve the problem to which the policy is responding.^19^ Borrowed from economics, the term ‘policy mix’ draws attention to how blending different policy instruments can affect intended policy outcomes in unexpected ways. The literature tends to depict failure to achieve outcomes as either due to inadequate policy design; or resulting from poor policy instruments’ interaction.^26^ We assessed Uganda’s adopted policy instruments in terms of their temporal stability (ie, over a fifteen-year period) and in terms of the extent to which they conform to what we introduce as the 3Cs: *coherence* – the synergies or logical linkage of policy instruments to the stated goal; their *comprehensiveness *– the completeness of instruments in regard to the three delaysand *consistency* – the absence of contradictions or the extent to which policy instruments work together towards the same goal.^19,31,35^ These relationships are shown in diagrammatic form below (see Figure 1).

###  Data Collection Methods 

 A retrospective mixed methods study was conducted at national level covering policies regarding maternal health. Uganda was treated as a single unit. Retrospective policy analysis was utilised as a powerful tool to inform future reform via reflection on past performance.^36^ Data were collected in two phases between April and July 2018. The first round involved document review triangulated by interviews with national policy-makers in the second round.

###  Documentary Analysis/Retrospective Policy Review 

 Documents reviewed were retrieved from the MoH Knowledge management portal (http://library.health.go.ug/publications) and the libraries of MoH, Makerere University and the Ugandan Parliament. Two main types of documents were assessed:

 (a) Maternal Health Policy Documents. A general search was conducted using the following strategy: “maternal health” AND “Reproductive Health” AND Policy OR Guidelines OR Standards OR Strategy OR Plan. The inclusion criteria were: (*i*) A document, which explicitly spelt out the objective of reducing maternal mortality; (*ii*) Covering the period 2000-2015, (*iii*) Authored by the government of Uganda. We excluded documents that did not directly state a policy on maternal health mortality and mapped 14 policies, goals, targets and interventions from 16 policy documents. Using a document review checklist, we extracted raw text on policy provisions, duration of the policy, goal, objectives/outcomes, targets and interventions/policy instruments for each of the three delays.

 (b) Government progress reports covering performance on various maternal health indicators in the MDG period. These included; Annual health sector performance reports (n = 11), Maternal and Perinatal Death Reviews (n = 3), UDHS (n = 4), Health Centre IV (HC IV) and Hospital Census (n = 1), and National Service Delivery Survey (n = 2). We extracted trend data for 15 years on the following indicators that reflect the three delays: access to maternal health services ie, population within 5 km to the nearest health facility (delay two), functionality of EmOC, stock levels of essential Reproductive Health (RH) medicines (delay three) and reference to any of the three delays in recorded maternal deaths (this could reflect any of the three delays). We then extracted corresponding narratives that helped interpret the statistics.

###  Interviews

 Key informant interviews (n = 6) were conducted with selected national respondents. These were supplemental and served to clarify our observations from document review. All participants had more than 10 years’ service and were involved in national maternal health policy processes between 2000 and 2015. They included: a researcher (n = 1), national maternal health programme managers (in service or retired) in the MoH (n = 2), an Obstetrician/Gynaecologist (n = 1) and consultants hired to develop or evaluate maternal health policies (n = 2). These brought vivid experiences from their direct involvement in policy design, implementation and evaluation to illuminate the observed performance with respect to the three delays. An interview guide was configured to elicit their perspectives on the different policy packages and what they thought influenced their performance. We obtained written informed consent from participants. All interviews were audio-recorded, transcribed verbatim and reviewed for consistency. Confidentiality was maintained by anonymising the transcripts using generic identifiers such as, “researcher, gynaecologist/ obstetrician and national programme manager.” All data was stored electronically under secret password protection only known by the corresponding author.

###  Analysis 

 The application of the 3Cs framework in assessing effectiveness of design is underpinned by the assumption that; (1) there is a clearly defined problem (in this case high maternal death due to the three delays), (2) the interventions/ policy instruments/solutions to alleviate the problem are known (in our case, the prescriptive interventions for the three delays outlined by Calvello et al,^37^ and, (3) there is a theory of change linking solutions to the problem – the three delay model.^11,38^ Then fidelity of design to the 3Cs was assessed by asking the questions; 1) do the policy instruments speak to the problem (Coherence)?, are all the policy instruments needed to alleviate the problem included (Comprehensiveness)? and, are the policy instruments mutually reinforcing/do they speak to each other (Consistency).

**Figure 2:**
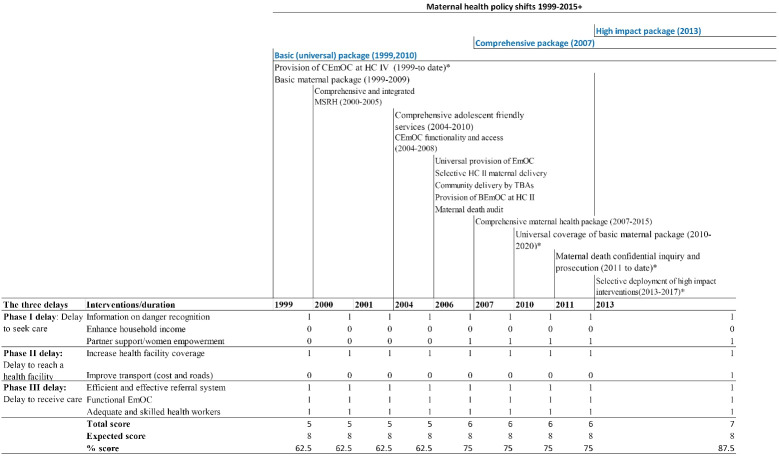
Testing the Comprehensiveness of 3D Policy Design. Abbreviations: CEmOC, comprehensive emergency obstetric care; MSRH, maternal sexual reproductive health; EmOC, emergency obstetric care; BEmOC, basic emergency obstetric care; TBAs, traditional birth attendants; HC IV, Health Centre level four. * Ongoing policies by the time of this study.

 We undertook a deductive manifest content analysis to assess policy design following three steps: Step 1) the extraction of policy instruments and interventions. Step 2) for each policy, interventions were grouped modelling the 3D framework. Step 3) each intervention/ instrument was tabulated against each of the 3Ds and mapped ( Tables S1 and S2, Supplementary file 1). Using our 3Cs framework for assessing policy mixes, a two-step analysis process was performed to assess policy design. Step 1: *comprehensiveness* was quantitatively assessed by assigning a score of 1 or 0 for the presence or absence of an instrument for each delay in comparison with prescriptive interventions for the three delays.^37^ The expected score was 8 to be able to conclude the policies put in place all the instruments to address the three delays. The total score was the actual score arising from the scoring of presence or absence of expected interventions. From the scores, proportions were computed by comparing aggregate scores per policy with the expected score of 8 across the MDG period (Figure 2) to observe a trend in comprehensiveness over the 15 years. Step 2: *Coherence* was assessed guided by the question; “do the instruments speak to the problem?” *Consistency* was assessed by answering the question; “are there observable contradictions or linkages among the policy instruments?” Interventions for each of the three delays as operationalised in a publication by Calvello et al^37^ were tabulated against interventions proposed in policy documents over the period 2000-2015. The corresponding author assessed through the surface (manifest) observation^39^ the presence and lack of coherence and consistency among policy instruments within and across the three delays over the 15 years being assessed. The presence or lack of coherence and consistency were colour coded green and red respectively (Supplementary file 2). The results were independently assessed using the same approach by three senior faculty close to the study and two senior faculty distal to the study based out of the Ugandan context. The corresponding author revised the table based on the feedback from the senior faculty and shared the final results which they approved of. Since interviews were few and focused on specific observations from the documentary review, we directly corroborated results from this with explanatory narratives from interviews as manually extracted and applied to enhance the interpretation of results conducted via our document review. To assess the manifestation of 3Ds we extracted data related to distance (population within 5 kilometres to the nearest health facility), functionality of EmOC, stock levels of essential RH and avoidable factors for maternal deaths. Illustrative quotes were extracted from the interviews to interpret and explain the results.

## Results

 Despite undertaking 14 policy shifts in the MDG period the 3Ds, which lead to maternal deaths, persisted. The shifts introduced were piecemeal and could only offer up 87.5% of all the policy instruments required to address the factors related to the 3Ds. There was observed absence of the 3Cs among the policy instruments notwithstanding the several policy shifts. Below we provide a detailed account of the prevalence of the 3Ds at the frontline and the loss of the 3Cs as policies were changing.

###  Mapping the Relevance of 3D Onto Uganda MDG

 According to the Uganda Maternal Death Review (MDR) report 2009-2011, Phase I delays in seeking care were reported in 70% of MDG maternal deaths. According to the UDHS report of 2011, this figure was attributed to inappropriate health-seeking behaviour. For example, in 2000, only 18.9% of women could spot the signs of pregnancy complication but this increased through policy reform to 51% in 2011. Although MDR reports 2009–2014 also attributed the delay I to the lack of familial support, the UDHS reports recorded it as a negligible factor. However, the delay in seeking care was attributed by key respondents (n = 2) to poor past experience of care.

*“We can put a lot of emphasis on community… most women now appreciate. But...they keep on dying within health facilities, eventually, they stop coming. To me, I would say one of the loopholes is we have failed to focus on those six pillars of the health system (service delivery, health workforce, health information systems, essential medicines, financing and leadership)*”[Obstetrician/gynaecologist].

 According to the Uganda Health Sector Strategic Plans for the period 2000-2015, the population within 5 km of the nearest health facility increased from 49% in 2000 to 72% in 2015.^40,41^ Due to this, we would expect to find deaths from Phase II delays declining. However, UDHS also shows that distance was still one of the major hindrances reported in 2000 by 44% of surveyed women (declining to 37.4% in 2015). Moreover, MDR recorded disparities in access to health facilities in rural areas. As such, lack of transport from home to the facility was found to be one of the frequent avoidable factors for maternal death:

*“This data suggests that the majority of women presented to the health facility rather late, and therefore had limited chances of survival especially in facilities that did not have 100% emergency readiness*”[MDR report 2012/2013, p. x].

 Strategies to mitigate deaths due to Phase II delays focussed on the selective prioritisation of geographical areas. For instance, the adopted 2006 maternal death notification and auditing policies covered less than a quarter (21.3%) of health facilities. According to the MDR report of 2014, the partograph used for monitoring labour was only used in 21% of the women who had a perinatal death.

 The MDR 2000-2014 records that Phase III delay (care received once at a health facility) was the number one factor in maternal deaths. For instance, 91% of the maternal deaths in health facilities during 2001 had been there between 2 hours and seven days (this figure reduced to 67% in 2014). These deaths were attributed to poor EmOC and inadequate referral systems. For example, the 2014 Census of Hospitals and HC IVs reported that not one Ugandan facility had all the items necessary to conduct deliveries with most (72%) operating at half the requirements. The annual health performance reports show that for two thirds of the MDG period health facility functionality to offer EmOC services was low at 23% while the availability of RH commodities and supplies was at 35%. Improvements were experienced in the final five years of the MDG period when availability of RH supplies increased to 64% and functionality of 45%. The MDR reported that health system deficiencies such as lack of resources, lack of transport between health facilities and inadequate staff training together accounted for between 50% and 65% of avoidable maternal death between 2012 and 2014. This corresponds to the inconsistencies observed in the policy decision to target only half of health facilities for EmOC in the same period.

*“…mothers were (often) referred from one hospital to another, yet all hospitals should provide Comprehensive Emergency Obstetric Care. This could have resulted in further delay of the women to receive care and consequently death. Although survival may depend on how critically ill one arrives at the facility, it is evident that most facilities were ill-prepared to save women with complications of pregnancy and childbirth”* [Extract from Maternal and Perinatal Death Reviews report 2009-2011, p. 27].

###  Mapping 3Cs Onto the 3D Framework

####  Assessing the Comprehensiveness of 3D Provision

 This study observed a general *lack* of *comprehensiveness* (completeness) regarding Phase I and II interventions with Phase III being the most comprehensively covered. The introduction of new policies alongside existing ones often meant that multiple policy instruments were in operation at the same time. For example in 2013, comprehensive and universal delivery of the basic package for maternal health co-existed with a new policy of high impact interventions in places experiencing high mortality.Attempts were made to enact a progressive build-up of interventions and we certainly saw comprehensiveness scores across the 3Ds increase from 62.5% in 2000 to 75% in 2006 before reaching 87.5% in 2013. However, while there was general progression towards comprehensiveness during the MDGs era, these policy shifts did not collectively provide the necessary interventions to cover all the three phases of delay. This is illustrated in diagrammatic form (see Figure 2).

####  Assessing the Coherence of 3D Provision

 When considering the *coherence *(synergies or logical linkage of policy instruments to the stated goal) of 3D policy implementation in MDG Uganda, we observed limited success in targeting Phase Irisk recognition. Between 2000 and 2005, facility-led policy instruments were introduced targeting information on obstetrics including goal-oriented ANC and postnatal care, adolescent health and birth preparedness. From 2006 there was an expansion of community-led policy initiatives including the establishment of village health teams. But a truly coherent approach to Phase I was never achieved since there were no instruments for most of the MDG period targeting financial barriers that hinder women from seeking care, securing the involvement of men and increasing women’s role in decision making regarding their own health.

 In terms of coherence of approach to Phase II delays, useful initiatives were introduced in relation to the availability of EmOC. Between 2000 and 2004 there was a heavy programme of construction and upgrading of existing health facilities. Yet lack of coherence was demonstrable because for twelve years (2000-2012) there were no policy instruments addressing transport to health facilities. A semblance of coherence here was only achieved in 2013 with the establishment of a public-private partnership for emergency ambulance services using a voucher system.

 With regards to Phase III delays, coherence was observed in several areas particularly with the introduction of new referral systems in 2000. Regarding the provision of EmOC, there was a coherent progression from putting in place services, improving quality of care and delivery management. However, there was still incoherence in terms of the shifting of tasks and provision of supplies to traditional birth attendants (TBAs) to provide delivery services in 2006 and the criminalisation of maternal death in 2011, which were not linked to quality improvement for EmOC. With respect to skilled providers of EmOC, coherence was observed in the build-up of policy instruments targeting recruitment, training and incentivising of health workers since 2000. Additional instruments on in-service training, staff accommodation and addressing restrictive staffing norms from 2004 strengthened synergies with existing policy instruments. Overall, however, the training of TBAs was largely incoherent, distorting synergies between skilled birth attendance, expansion of health facilities and roll out of EmOC:

*“*You see TBAs as much as we skilled them, they could not provide care for the complications and yet those complications were killing mothers and these TBAs used to delay mothers and make late referrals*” *[MoH retired official].

####  Assessing the Consistency of 3D Policy Design

 Inconsistencies (contradictions) in policy instrument design was observed across each of the three phases of delay. The concurrent operation of basic, comprehensive, universal and selectively targeted policies created lack of synergy. In 2004, for example, adolescent health information was introduced, dropped in 2010 and reintroduced in 2013. Policy instruments were consistent in terms of the expansion of health facilities from 1999 to 2004 but inconsistencies were introduced in 2006, 2010 and 2013 as tensions between universal, basic, comprehensive, and high impact policies were exposed. The 2006 focus on rolling out of health facilities in hard to reach and high burden areas, for example, was contrary to the commitment to universal coverage. Similarly, while pursuing universal coverage, the introduction of policy instruments to deliver adolescent-friendly services across only three quarters (75%) of health facilities, the provision of accessible health facilities to 50% of the mobile population and the functionalizing of 50% of HC IVs in 2010 meant that significant numbers of people were still being left out of provision. Inconsistencies were further entrenched in 2013 with the high impact policy instruments targeting implementation in certain areas (Karamoja, southwest, western, eastern and northern regions) whilst other instruments focused on scaling up of EmOC in all areas.

*“The health system does not have the ability to implement these working solutions to scale so we still regionalize, pilot and if we are to scale up, we scale to 20 or 30 districts. Even the current (transport) voucher project [...] just picked a few districts to implement that so when we are really ever going to scale”* [Senior MoH official_01].

 Furthermore, the continued establishment of HC IIs was observed to be inconsistent with the pace of functionalising them.

*“There has been a mismatch between the construction of HC IIs countrywide and the speed at which resources are made available for their operationalization with new facilities remaining closed for lack of staff, basic equipment and drugs. The construction has also been geographically inequitable. The increase in funding for drugs has not been enough to prevent frequent stockouts in health facilities”* [Excerpt, MoH 2004, Health Sector Strategic Investment Plan II, p. 6].

 Phase III delay policy instruments were generally inconsistent. While policy instruments addressing referral and provision of EmOC between 2000 and 2004 appeared consistent, the introduction of TBAs as skilled providers contradicted EmOC provision that emphasised midwifery skills, supplies and equipment. In addition, the decision to avail skilled providers for only 50% of deliveries meant that this policy was not designed to offer skilled providers for all expected deliveries. There are many other examples available. For instance, in 2006, while EmOC was being rolled out to HC II, ambulances were available at only 85% of HC IVs. HC II and III, which are the first to receive patients, were only given motorcycles and bicycles for transport. With regard to the provision of EmOC, policy instruments were divergent. For example, EmOC was to be rolled out from HC III onwards, but at the same time, it was to be rolled out from HC II onwards but only in HC II located in areas with limited access to EmOC. When it came to equipping health facilities for EmOC, policy instruments only provided for 50% of HC IVs and 42% of hospitals while HC II and III were left out. Commenting on these inconsistencies, one of our respondents noted:

*“When you go into details, you realize the main causes of maternal death were the same. So why didn’t we just come up with a policy addressing the causes of maternal death to guide implementation?” *[Policy expert hired by MoH].

## Discussion

 This study disentangled the various constituent parts of policy design using a framework of 3Cs constructed from combing 3D and policy mixes to conduct a systematic analysis of effectiveness in policy design. The results from the analysis show that the policy instruments failed to effectively address the three delays that cause maternal deaths. This emanated from poor policy design that was characterised by contradictions, incompleteness and disjointedness among the policy instruments. We suggest that the 3Cs framework is a useful tool for informing the policy design process to guarantee better policies.

 We observed clear tensions in the design of the various packages of policy implemented during Uganda MDG. This may not surprise. The use of ‘basic packages’ (also known as Essential Healthcare Packages or Minimum Health Care Packages), for example, arose out of selective 1980s primary healthcare debates^42^ before being pushed by the World Bank in 1993.^43^ These interventions tend to focus on cost-effective policies that significantly reduce financial burdens through minimising the services that target key drivers of morbidity and mortality. Comprehensive policy packages, conversely recommend a rather expensive or holistic package for all. However, these can be criticised as being unattainable in low resource settings such as Uganda’s.^44^ Similarly, high impact packages^45,46^ or “quick win-quick-impact, life-saving or priority interventions” were by design targeted and derived from conversations that sought to forestall the likelihood of missing MDG 5.^47-51^ Therefore policy designs aligned to the basic (universal), comprehensive and high impact packages each had several limitations.^48,52^ These limitations presented complex challenges for achieving harmonised policy design. In the Ugandan case, it appears that the repeated failure by policy to achieve MDG 5 arose from unsuccessful attempts to reconcile what were, in fact, irreconcilable basic, comprehensive, and high impact policy packages. Attempts to navigate from one package to another rendered policies both inefficient and ineffective.^53^ Given that Uganda was facing multiple morbidities, the minimum package could not fit within the available resources leading to rationing of interventions without significant impact. Similar experiences were reported elsewhere where high impact initiatives achieved only a third of their targets.^54^ Indeed studies conducted in Uganda and Zambia suggest that independently addressing any of the 3Ds produces positive impact, but it is not sufficient to impact maternal mortality.^55-59^ From Rwanda’s experience, effective policy design requires carefully selected policy instruments cutting across 3D in optimal combinations to target immediate, medium and long term maternal mortality.^60^

 The incoherencies and inconsistencies observed among policy instruments across different policy shifts notwithstanding the failure to achieve the policy goal of reducing maternal mortality over years are typical of policy layering. Policy layering occurs when new policies are introduced without discarding or aligning with existing ones targeting the same goal.^61^ Similar to the analysis of evolutions in British food policy, incremental policy change observed in Uganda’s maternal health policies characterised by the concurrent operation of basic, universal, comprehensive and high impact policies created a complex policy mix producing instruments that were not mutually supportive.^62,63^ This differs from findings from other studies which contend that incremental policy layering can result in durable, resilient and successful policy reforms.^64^

 Our findings suggest that a 3Cs approach can be successfully applied as a framework to assess the effectiveness of policy design. This can be done by adopting a causal theory underlying the design of a policy and applying the 3Cs framework to systematically assess effectiveness of design. In this study, the three-delay model was adopted as the causal theory underlining the policy shifts. Since there is limited research on policy design for maternal health, we drew on studies evaluating policy intervention more generally. The necessity of linking interventions across the 3Ds must be emphasised. By applying the 3Cs framework, we demonstrate that despite incremental policy change over fifteen years of the MDGs, policy shifts failed to achieve a comprehensive package of mutually reinforcing and linked policy instruments to achieve the targeted reduction in maternal mortality. Like other studies, we underscore the fact that incremental policy change should build up towards Comprehensiveness but they must also maintain Consistency and Coherence to achieve optimal performance.^26,65-69^ Contradictions have also been cited in task shifting – defined as “delegating tasks to existing or new cadres with either less training or narrowly tailored training,”^70^ which has been described as a policy developed without clear guidance for implementation.^71^ Policy designs supporting selective introduction of policy instruments have also been described as ineffective and disruptive to the realisation of the goal.^48^ Our findings align with other analyses of performance of maternal health policies which recommend matching policy instruments across all the delays in the policy design to realise reduced maternal mortality.^72^ This is further supported by an evaluation of the persistence of demand and supply gaps for maternal health services in Uganda which on finding all the three delays prevalent, recommended adoption of an appropriate instrument mix covering the three delays in the policy designs.^73^ The prevalence of the 3Ds in recorded maternal deaths can be observed at policy implementation.^74^ Moreover, we contend that maternal deaths due to any of the three delays can be traced back to ineffective policy design.^9^ There are several accounts in literature to illustrate the lack of linkages between the 3Ds and 3Cs in maternal health policy implementation.^75^ Lack of awareness of obstetric risks and poor birth preparedness among women have been reported in Uganda, for example. This has been attributed to incomplete intervention packages in policy design.^76,77^ This is supported by our findings which show that such policy instruments for the first six years of the MDG period targeted women while excluding family members (like men) who significantly influence care-seeking decisions. Similar findings were reported in Tanzania and Uganda. Tanzanian men reported that they did not facilitate care seeking because of lack of information on maternal care^78^ while Ugandan men considered pregnancy and childbirth as a women’s matter.^79^

 It is however worth recognising that maternal mortality is a composite outcome of several factors within and outside the health sector.^3^ Some of these are reflected in the three delay model especially concerning the first and second delays particularly poverty, social-cultural factors, gender dynamics and transport. But, there are also other broader issues that affect maternal health including lack of political support, funding, access to medical technologies, poverty, religious beliefs, exposure to conflict, education, urbanisation and many other factors. Several studies have reported these issues in Uganda. For example, lack of political priority for maternal health services at the primary healthcare level was reported to affect the quality of care.^80^ Ultrasound scan equipment was reported to be inaccessible in rural areas without access to electricity.^81^ Low urbanisation, religious values and poverty have been reported to hamper access to maternal health services with women from poor households and residing in rural settings finding difficulties in accessing the desirable maternal packages.^82^ On the other hand, women affected by political conflict in Northern Uganda and Burundi faced difficulties accessing maternal health services.^83^ Finally, maternal mortality has also been reported to be higher among women with low formal educational attainment.^84^

 Therefore, while in our analysis and indeed our proposition of the 3Cs framework in this study we apply the three-delay model as an underlying theory of change, we are cognisant that they are not the only factors at play in maternal mortality and the failure to achieve targets. Rather, we use the three-delay model to signal to scholars and practitioners of policy analysis who may apply the 3Cs framework in future the core principles underpinning it. Namely; (1) defining the problem clearly, (2) assembling known interventions/ policy instruments/solutions to alleviate the problem, (3) linking solutions to the problem using a theory of change thinking,^11,38^ and, (4) aligning the policy design to ensure that; the interventions speak to the problem (Coherence), all the solutions needed to alleviate the problem are included (Comprehensiveness) and the solutions are mutually reinforcing/they are speaking to each other (Consistency).

## Conclusion

*“The sector made significant progress […] but, it’s unlikely to achieve targets for Maternal Mortality Ratio. […]. The three classic delays (home, on the way, and at the facility) must be addressed to reign in on the unacceptably high maternal morbidity and mortality”* [MoH, Annual Health Performance Report 2014/2015. p. 4].

 The repeated failure to achieve maternal health targets during the MDG period despite multiple policy shifts and policy packages can be linked to the absence of the 3Cs in policy design. Given that the various shifts did not deliver an exhaustive package of instruments to effectively reduce maternal mortality, it appears the policy changes were not achieving optimal balance. Policies accumulated incrementally and did not guarantee provision for all necessary interventions. The design of policy was ineffective. Given that the SDG period inherited some MDG policies, there is a need to revisit all the policies and aggregate them into one major policy approach that outlines the “must have” instruments that address all the 3Ds. The design of subsequent policy reforms should be carefully structured to achieve a unified theory of change to maintain synergies across policy transitions. Future research should consider exploring the drivers of policy reforms in maternal health and developing contextual intervention packages to holistically address the 3D. Based on the results from this study, we suggest that using the 3Cs framework is helpful in assessing the effectiveness of policy design particularly with regard to the 3Ds. Reflecting on maternal health policy changes in Uganda during MDGs, our study shows that a lack of comprehensiveness, coherence and consistency among policy instruments contributed to the failure to achieve MDG 5.

###  Strengths and Limitations

 This study was informed by nationally representative policy documents including routine programme reports, population-based surveys and a census. Documentary data were triangulated with interviews involving national policy-makers and programme implementers. Given that, this study has a national scope, it provides only a general national outlook. Although the 3Cs framework provides immense analytical value for assessing effectiveness of policy design, validating it across programmes, sectors and contexts would further increase its utility. By applying the three-delay model, this study does not reflect on the other factors affecting maternal mortality that could account for failure to achieve targets. However, we have articulated the principles underpinning our analysis and discussed them in the context of the broader literature on other determinants of maternal health to enhance the utility of our findings and proposed framework. The approach of surface observation used in assessing coherence and consistency could be subject to observation bias. This was minimised by subjecting the analysis to independent review by senior members of faculty both close and distal to the study.

## Acknowledgements

 We are grateful to the national policy-makers in Uganda who participated in this study. We acknowledge the German Academic Exchange Service (DAAD) for the partial doctoral scholarship and the Erasmus*Plus* mobility programme of Nottingham Trent University for the writing residency support.

## Ethical issues

 This study was approved by the Higher Degrees Research and Ethics Committee of the School of Public Health, Makerere University and cleared by the Uganda National Council for Science and Technology (Ref: SS 4484). All participants interviewed provided written consent. Interviews and quotes were anonymised.

## Competing interests

 Authors declare that they have no competing interests.

## Authors’ contributions

 MM with the guidance of FS and SNK conceptualized and designed the study. MM participated in data collection. MM guided by FS, SNK, and GJ conceptualised the paper. MM and GJ performed the analysis and drafted the paper. FS, LG, SNK, and DM reviewed the draft manuscript and provided significant intellectual input leading to several revisions. All authors reviewed and approved the final manuscript.

## Disclaimer

 The views expressed in this article are for the authors and not the position of the institutions of affiliation or the funders.

## Funding

 This study was jointly funded by the Health Policy Analysis Fellowship programme, supported by the Alliance for Health Policy and Systems Research, Switzerland, and the SPEED Project – a project at Makerere University School of Public Health focusing on evidence generation to inform health policy-making in Uganda.

## Supplementary files

Supplementary file 1 contains Tables S1 and S2.

Supplementary file 2. Assessment of Coherence and Consistency Across Policy Shifts.
