# Supplementary material for: Designed to Fail? Revisiting Uganda’s Maternal Health Policies to Understand Policy Design Issues Underpinning Missed Targets for Reduction of Maternal Mortality Ratio (MMR): 2000-2015
Source: Int J Health Policy Manag. 2021 Sep 7;11(10):2124–34. doi: 10.34172/ijhpm.2021.127 (PMC9808297; doi:10.34172/ijhpm.2021.127)
Supplement: Supplementary file 1 — contains Tables S1 and S2. [file ijhpm-11-2124-s001.pdf]

**Article title:** Designed to Fail? Revisiting Uganda's Maternal Health Policies to Understand Policy Design Issues Underpinning Missed Targets for Reduction of Maternal Mortality Ratio (MMR): 2000-2015

**Journal name:** International Journal of Health Policy and Management (IJHPM)

**Authors' information:** Moses Mukuru[1]*, Jonathan Gorry[2], Suzanne N. Kiwanuka[1], Linda Gibson[2], David Musoke[3], Freddie Ssengooba[1]

[1]Department of Health Policy, Planning and Management, School of Public Health, College of Health Sciences, Makerere University, Kampala, Uganda.

[2]School of Social Sciences, Nottingham Trent University, Nottingham, UK.

[3]Department of Disease Control and Environmental Health, School of Public Health, College of Health Sciences, Makerere University, Kampala, Uganda.

(*Corresponding author: mmukuru@musph.ac.ug)

**Supplementary file 1.**

**Table S1: National maternal health policy documents reviewed**

| No | Document description | Type | Year/ lifespan |
|---|---|---|---|
| 1. | GoU, (1999), Health sub district policy; Roll out of CEmOC within 5KM radius of the population | Health sector policy | 1999- to date |
| 2. | MoH, (1999), National Health Policy - Essential antennal and Obstetric care | Health sector policy | 1999-2009 |
| 3. | MoFPED, (2000), Uganda's Poverty Eradication Action Plan; Reduction of maternal death as an essential strategy of poverty reduction | Overall national policy framework | 2000 - 2004 |
| 4. | MoH, (2000), Health Sector Strategic Plan 2000/01 – 2004/05; maternal health as an essential element of comprehensive and integrated reproductive health | Health sector strategic plan | 2000 - 2005 |

| No | Document description | Type | Year/ lifespan |
|---|---|---|---|
| 5. | MoH, (2001),The National Policy Guidelines and Service Standards for Reproductive Health Services; Quality integrated reproductive health service | Programme guidelines | 2001 - 2004 |
| 6. | MoH, (2004), National Adolescent Health Policy for Uganda; introduction of post abortion care and adolescent friendly services | Programme Policy | 2004 |
| 7. | MoFPED, (2004), Uganda's Poverty Eradication Action Plan; Increasing access to functional CEmOC | Overall national policy framework | 2004 - 2008 |
| 8. | MoH, (2006), The National Policy Guidelines and Service Standards for Sexual and Reproductive Health and Rights 2006; TBA as skilled providers, Provision of BEmOC at HC II, maternal and perinatal mortality auditing | Programme guidelines | 2006 |
| 9. | MoH, (2005), Health Sector Strategic Plan II 2005/06 – 2009/2010; Universal delivery of the Uganda National Minimum Health Care Package, Selective provision of delivery services at HC II, Maternal death reviews | Health sector strategic plan | 2006 - 2010 |
| 10. | MoH, (2007), Roadmap for Accelerating the Reduction of Maternal and Neonatal Mortality and Morbidity in Uganda; Holistic approach to delivery of maternal health services (using the three delay model) | Programme Strategy | 2007 - 2015 |
| 11. | MoH, (2010), The second national health policy; Universal Access to EmOC and MH services | Health sector policy | 2010 - to date |
| 12. | NPA, 2010), National Development Plan (2010/11 - 2014/15); Health systems and quality of care for maternal health | Overall national policy framework | 2010 - 2015 |
| 13. | MoH, (2010), Health Sector Strategic & Investment Plan 2010/11 – 2014/15; Universal Coverage of maternal and other services, Institutionalise delivery at HC II | Health sector strategic plan | 2010 - 2015 |

| No | Document description | Type | Year/ lifespan |
|---|---|---|---|
| 14. | MoH, (2011), The National Adolescent Health-Strategy; Comprehensive Adolescent friendly SRH | Programme Strategy | 2011-2015 |
| 15. | Ministerial directive on Maternal death review (Admin.45/273/01 dated 10th May 2011); Confidential inquiry and criminal prosecution | Ministerial directive | 2011 to date |
| 16. | MoH, (2013), Reproductive Maternal, New-born and Child Health Sharpened Plan for Uganda; High impact lifesaving interventions for women | Programme Strategy | 2013 - 2017 |

## Table S2. National maternal health programme performance reports reviewed

| No | Document description | Type | Year/ lifespan |
|---|---|---|---|
| 1. | Annual Health Sector Performance Report Financial Year 2000/2001 | Health sector programme report | 2001 |
| 2. | Uganda Bureau of Statistics (UBOS) and ORC Macro.2001. Uganda Demographic and Health Survey 2000-2001. Calverton, Maryland, USA: UBOS and ORC Macro. | National survey report | 2001 |
| 3. | Annual Health Sector Performance Report 2003/2004 | Health sector programme report | 2004 |
| 4. | Annual Health Sector Performance Report 2004/2005 | Health sector programme report | 2005 |
| 5. | Uganda Bureau of Statistics (UBOS) and Macro International Inc. 2007. Uganda Demographic and Health | National survey report | 2007 |

| No | Document description | Type | Year/ lifespan |
|---|---|---|---|
| | Survey 2006. Calverton, Maryland, USA: UBOS and Macro International Inc. | | |
| 6. | Annual Health Sector Performance Report 2006/2007 | Health sector programme report | 2007 |
| 7. | Uganda National Service delivery report 2008 | National survey report | 2008 |
| 8. | Annual Health Sector Performance Report 2008/2009 | Health sector programme report | 2009 |
| 9. | Annual Health Sector Performance Report 2009/2010 | Health sector programme report | 2010 |
| 10. | Annual Health Sector Performance Report 2010/2011 | Health sector programme report | 2011 |
| 11. | Maternal & Perinatal Death Reviews - report Uganda: 2009-2011 | Health sector programme report | 2011 |
| 12. | Annual Health Sector Performance Report 2011/2012 | Health sector programme report | 2012 |
| 13. | Uganda Bureau of Statistics (UBOS) and ICF International Inc. 2012. Uganda Demographic and Health Survey 2011. Kampala, Uganda: UBOS and Calverton, Maryland: ICF International Inc. | National survey report | 2012 |
| 14. | Annual Health Sector Performance Report 2012/2013 | Health sector report | 2013 |

| No | Document description | Type | Year/ lifespan |
|---|---|---|---|
| 15. | Maternal and Perinatal Death Review Uganda 2012/13 | Health sector report | 2013 |
| 16. | Annual Health Sector Performance Report 2013/2014 | Health sector programme report | 2014 |
| 17. | Health centre IV/Hospital census | National Census report | 2014 |
| 18. | Annual Health Sector Performance Report 2014/2015 | Health sector programme report | 2015 |
| 19. | Maternal and Perinatal Death Review report - Uganda 2013 /14 | Health sector programme report | 2015 |
| 20. | Uganda National Service delivery report 2015 | National survey report | 2015 |
| 21. | Uganda Bureau of Statistics (UBOS) and ICF. 2018. Uganda Demographic and Health Survey 2016. Kampala, Uganda and Rockville, Maryland, USA: UBOS and ICF. | National survey report | 2018 |
